# The Effect of the Use of Unconventional Solutions for Osmotic Dehydration on Selected Properties of Fresh-Cut Oranges

**DOI:** 10.3390/foods14030468

**Published:** 2025-02-01

**Authors:** Sabina Galus, Katarzyna Rybak, Magdalena Dadan, Dorota Witrowa-Rajchert, Małgorzata Nowacka

**Affiliations:** Department of Food Engineering and Process Management, Institute of Food Science, Warsaw University of Life Sciences, 159c Nowoursynowska St., 02-776 Warsaw, Poland; katarzyna_rybak@sggw.edu.pl (K.R.); magdalena_dadan@sggw.edu.pl (M.D.); dorota_witrowa_rajchert@sggw.edu.pl (D.W.-R.)

**Keywords:** osmotic dehydration, orange, polyols, fruit juice, fruit concentrate, physicochemical properties, organoleptic assessment

## Abstract

This study investigated the effects of unconventional solutions on the osmotic dehydration of oranges. These solutions included xylitol, fruit concentrates (strawberry, cherry, orange), rosehip juice, and sucrose. The study examined dehydration kinetics, dry matter, total soluble solids, water activity, color, texture, sugars, vitamin C, polyphenols, carotenoids, and antioxidant potential, alongside microstructural observations. The results indicated that osmotic solutions and the dehydration time (3 h) significantly influenced the oranges’ physical and chemical properties. Cherry and strawberry concentrate solutions caused the greatest color changes, enhancing the dried product’s visual appeal. Oranges dehydrated with strawberry concentrate exhibited the highest polyphenol content (2909 mg chlorogenic acid/100 g d.m.) and antioxidant potential (11.0 mg TE/d.m.), while rosehip solution yielded the highest vitamin C levels (80.27 g/100 g d.m.), followed by strawberry (62.32 g/100 g d.m.) and orange (47.67 g/100 g d.m.) concentrates. These findings highlight the benefits of using fruit concentrates and juices in osmotic dehydration. The unconventional osmotic solutions resulted in a reduction in the hardness of dehydrated orange sliced from 0.65 N to the range of 0.36–0.60 N, except for strawberry concentrate, which resulted in the highest value (0.72 N). Key parameters, such as the water activity, dry matter, and dehydration efficiency, were more favorable compared to those in the sucrose solution samples. The organoleptic assessment recommended xylitol for maintaining sweetness without altering taste or smell, whereas strawberry juice scored lowest due to its foreign taste and smell. Overall, osmotic dehydration enhanced the nutritional and sensory attributes of oranges by allowing the penetration of bioactive compounds, making them superior to fresh raw material in tested parameters.

## 1. Introduction

Oranges are members of the citrus genus of the *Rutaceae* family, which are distinctive, widely consumed fresh fruits particularly appreciated for their pungent flavor [1]. Their pulp is an excellent source of vitamin C, providing 64% of an individual’s daily requirement. In addition to vitamin C, oranges are rich in folic acid and potassium, and are an excellent source of bioactive antioxidant ingredients [2,3]. Oranges are citrus fruits grown in warm climates and are a source of vitamin C, B vitamins, and provitamin A. Like other citrus fruits, they are considered a source of bioactive compounds such as ascorbic acid, carotenoids, flavonoids, and phenolic compounds, all of which are considered to promote health [4,5,6]. In addition to fresh fruit, oranges are available in the form of juices, jams, preserves, marmalades, and jellies [7]. Moreover, the application of orange waste improves the nutritional quality of different foods, such as bakery products, offering interesting sustainability benefits. The valorization of orange waste opens new routes for food product development and new natural ingredients for the food industry [2]. However, the greatest health benefits come from fresh oranges which, unfortunately, as perishable fruit, last for only a few days. The main cause of fruit and vegetable spoilage is their high water content [8]. To increase their durability, many methods have been tried, and osmotic dehydration is one of the most suitable ones.

Osmotic dehydration is widely used to partially remove water from plant tissues by immersing them in a hypertonic (osmotic) solution [9]. In this process, the driving force is the higher osmotic pressure of the hypertonic solution, which causes water to diffuse from the tissue into the solution [10]. At the same time, the membrane responsible for osmotic transport is not perfectly selective, meaning that other solutes present in the cells may also be washed out into the osmotic solution [11]. Osmotic dehydration is an important process in many food materials that aims to reduce water activity to inhibit microbial growth [12].

Most foods contain large amounts of water, making them expensive to transport, package, and store [13]. Osmotic dehydration is considered an energy-efficient method of partial dehydration because it does not require additional energy to convert the product from a liquid to a solid state [14,15]. One of the most commonly used osmotic solutions is sucrose solution, which has been used in the production of many food products, including dried and candied fruits and vegetables [16]. Recent studies have shown that the combination of various osmotic agents is more effective in comparison to sucrose alone due to the mixture of solute properties [17,18].

An interesting group of new osmotic agents are polyols, usually obtained through the chemical reduction of saccharides such as xylitol, sorbitol, erythritol, maltitol, lactitol, mannitol and isomalt [19]. In general, polyols have a sweet flavor and physical properties similar to those of sugar, making them effective as low-calorie sweeteners [20]. Additionally, polyols have a lower caloric value than sucrose due to their poor absorption in the gut, which can benefit people who struggle with diabetes or obesity. Moreover, polyols do not contribute to caries formation as they are not metabolized by the oral microbes associated with dental plaque [21]. Xylitol and erythritol were found to be suitable as osmotic agents in the dehydration of apples, making these solutions an alternative to sucrose. However, solutions containing maltitol, inulin, or oligofructose were ineffective [22]. Moreover, using polyols in osmotic dehydration may cause changes in the sensory attributes of prepared snacks. Konopacka et al. [23] observed that each of the investigated osmotic solutions significantly influenced the taste and texture profile of the dehydrated fruit, affecting their sensory acceptability. The changes were dependent on the fruit species and the drying method used. In this context, using different osmotic solutions may play a crucial role in the design of new food products with a controlled color or nutritional value.

Fruit juices or concentrates are good examples of new osmotic agents that make it possible to create products with an enhanced bioactive composition [24]. Fruit or vegetable juice concentrates contain sugars, mainly fructose and glucose, and other native substances with different molecular sizes. Using them as agents for osmotic dehydration while enriching plant materials creates a unique taste and usually affects color [25]. Concentrates with a soluble solid content of about 70% are obtained from fresh or frozen fruits or vegetables without the addition of chemical preservatives [26]. Dried fruits pre-treated with fruit juice have a predominantly sweet taste, while those treated with more sour concentrated juice may exhibit high acidity. Therefore, the study aimed to determine the effect of unconventional osmotic solutions made from xylitol, fruit concentrates from strawberries, cherries and oranges, and rosehip juice compared to sucrose on selected physical and chemical properties of dehydrated orange slices, as well as on their organoleptic properties. The dry matter content, water activity, total extract content, color and texture parameters, sugar and vitamin C content, polyphenol content, anti-radical potential (with ABTS radical and iron III reduction), and carotenoids were examined. The structure of the dried samples after the osmotic dehydration process was analyzed based on photographs taken using a scanning electron microscope.

## 2. Materials and Methods

### 2.1. Experimental Conditions, Protocol and Materials

The research material consisted of Navelina oranges from Spain, purchased on the Polish market, and harvested in the 2022 season. All experiments and analyses were performed in the laboratories of the Department of Food Engineering and Process Management at Warsaw University of Life Sciences (Poland).

The characteristics of the fresh fruit are presented in Table 1. The fruit was characterized by a relatively low dry matter content (14.55%) and high water activity (0.942), typical of fresh raw materials. The color parameters were L* = 49.59 (lightness), a* = 1.67, and b* = 22.78, respectively, indicating a significant share of yellow. The fruits were also characterized by a soft texture (0.65 ± 0.05 N) and low sugar content, but a high content of polyphenols (2527 ± 63 mg chlorogenic/100 g d.m.) and vitamin C (42.73 ± 0.22 mg ascorbic acid/100 g d.m.).

The fruit was washed in a 1% sodium hypochlorite solution (Avantor Performance Materials Poland S.A., Gliwice, Poland), then cut into 5 mm slices using a manual mandoline slicer with adjustable thickness. The slices were subjected to an osmotic dehydration process in selected osmotic solutions: sucrose, xylitol, cherry concentrate, strawberry concentrate, orange concentrate, and rosehip juice. To carry out chemical and structural analyses, the tested material was preserved using the freeze-drying method. The material was pre-frozen to −40 °C using a shock freezer (Shock Freezer HCM 51.20, Irinox, Treviso, Italy) and then dried in a freeze dryer (Gamma 1-16 LSC, Martin Christ Gefriertrocknungsanlagen GmbH, Osterode am Harz, Germany) at a shelf temperature of 30 °C, a pressure of 63.0 Pa, and a condenser temperature of −55 °C for 48 h. The obtained material was placed in airtight PET/AL/PE bags (Pakmar, Warsaw, Poland. Before chemical analyses, the material was ground in an IKA grinder (model A11 basic, IKA-Werke GmbH & Co. KG, Staufen im Breisgau, Germany).

### 2.2. Technological Methods

#### 2.2.1. Preparation of Osmotic Solutions

All osmotic solutions were prepared by dissolving a specific mass of substance or fruit juice concentrate in distilled water. The concentration of all obtained osmotic solutions was 50 °Brix, except for the rosehip juice solution, which also contained trehalose (Hortimex, Warsaw, Poland) to achieve the desired concentration.

#### 2.2.2. Osmotic Dehydration

The osmotic dehydration method and conditions were used based on previous experiments for different fruits and vegetables and a preliminary study using orange slices with different thicknesses. The equipment available for stabilizing temperature and stirring was used to optimize the experiments. The osmotic solutions were poured into 1 L beakers and placed in a water bath equipped with a VSLB18 mixing system (VWR International, Radnor, PA, USA). Next, 50 g of the previously prepared raw material and the osmotic solution were placed in each beaker. The mass ratio of the solution to the samples was 1:4. This process was carried out at a constant temperature of 30 °C with stirring at 100 rpm for 3 h.

The kinetics of the dehydration process were measured in triplicate after 0, 0.5, 1, 2, and 3 h, and were based on the mass loss Δ*M_t_*° (kg/kg), water loss Δ*M_t_^w^* (kg H_2_O/kg orange), and the increase in dry matter mass Δ*M_t_^ST^*, which were calculated using the following formulas:

(1) $${{∆M}_{t}}^{{}^{\circ}}={M_{t}}^{{}^{\circ}}-{M_{0}}^{{}^{\circ}}=\frac{m_{t}-m_{0}}{m_{0}},$$

(2) $${{∆M}_{t}}^{w}={M_{t}}^{w}-{M_{0}}^{w}=\frac{m_{t}x_{wt}-m_{0}x_{w0}}{m_{0}},$$

(3) $${{∆M}_{t}}^{\mathrm{ST}}={M_{t}}^{\mathrm{ST}}-{M_{0}}^{\mathrm{ST}}=\frac{m_{t}x_{STt}-m_{0}x_{ST0}}{m_{0}},$$

where *M*_0_—initial weight of oranges before the dehydration process [kg]; *M_t_*—final weight of oranges [kg]; *x_w_*_0_ and *x_wt_*—mass of water before and after the dewatering process [kg/kg]; and *x_ST_*_0_ and *x_STt_*—mass of dry substance before and after the dehydration process [kg/kg].

### 2.3. Analytical Methods

#### 2.3.1. Dry Matter Content

The dry matter content was determined in fresh oranges and after the osmotic dehydration process using the drying method at 70 °C for 24 h, using a laboratory dryer SUP-65 WG (WAMED S.A., Warsaw, Poland). The percentage of dry matter in the tested material was calculated using the difference in weight before and after drying. The assay was performed in quadruplicate for each sample.

#### 2.3.2. Water Activity

The water activity was determined in fresh material and after the osmotic dehydration process using an AquaLab apparatus with an accuracy of ±0.003 (Series 3 model TE, Decagon Devices Inc, Pullman, WA, USA) following the manufacturer’s instructions. The measurements were performed in triplicate at a temperature of 23 ± 1 °C.

#### 2.3.3. Total Soluble Solids

The analysis of the total soluble solid content in oranges was performed using the refractometric method by squeezing the juice from the dewatered material. The measurement was performed using a refractometer (PAL-3, Atago Instruments, Tokyo, Japan). The results were obtained in °Brix. The assay was performed in triplicate.

#### 2.3.4. Color

The reflectance method in the CIE L*a*b* system was used to measure orange color parameters using a CR-400 colorimeter (Konica Minolta, Tokyo, Japan). Measurements were performed with the following settings: standard observer 2°, D65 light source, and a measurement diameter of 3 mm. The colorimeter was calibrated using a white reference plate with constant L*a*b* values. Color changes were determined in the fresh material before and after the osmotic dehydration process. Ten color measurements were made for each sample. Based on the obtained results, the absolute color difference (ΔE) was calculated using the following formula:

(4) $$∆E=\sqrt{{\left(∆L*\right)}^{2}+{\left(∆a*\right)}^{2}+{\left(∆b*\right)}^{2}},$$

where L*—lightness; a*—chromatic coordinate that determines the red (+) and green (−) colors; b*—chromatic coordinate that determines the yellow (+) and blue (−) colors; and ΔL*, Δa* and Δb*—color differences measured for osmotically dehydrated oranges and fresh oranges.

#### 2.3.5. Texture

The texture of the samples was measured using a TA-TX2i texture analyzer (Stable Micro Systems, Surrey, UK) at a temperature of 20 ± 2 °C. A compression test was performed to determine the maximum work and compression force of the orange. The compression force was generated by the Texture Export computer program (Windows). The test was performed at a constant compression speed of 20 mm/min until 50% deformation of the initial height of the material was achieved. Ten measurements were made for each sample.

#### 2.3.6. Determination of Sugar Content

The sugar content was determined using the UPLC-PDA system (WATERS Acquity H-Class, Milford, MA, USA) with refractive index detection [18]. The material was crushed and then weighed on an analytical scale of 0.2–0.3 g and poured with distilled water at a temperature of 80 °C. The prepared samples were placed in a circular vibrating shaker, and extraction was carried out for 4 h. The solution was then centrifuged (5 min, 6000 rpm), filtered (0.2 µm PTFE syringe filter), and injected into the column (injection volume 10 µL). The analysis of the sugar content in the tested material was carried out under isocratic conditions. The column temperature was 90 °C, the detector temperature was 50 °C, and the flow rate of the mobile phase, which was redistilled Milli-Q water, was 0.6 cm^3^/min. Quantitative analysis was performed based on the calibration curves prepared for sucrose, glucose, and fructose standards (Sigma-Aldrich, Steinheim, Germany). The assay was performed in duplicate.

#### 2.3.7. Determination of Vitamin C Content

The vitamin C content in the material was determined using the UPLC-PDA system (WATERS Acquity H-Class, Milford, MA, USA) [18]. The dried material was ground (IKA A11 basic, IKA-Labortechnik, Staufen, Germany), weighed (0.2–0.3 g), and mixed with 10 cm^3^ of cooled extraction reagent—3% metaphosphoric acid, 8% acetic acid (VWR Chemicals BDH Prolabo, Leuven, Belgium). The sample was then mixed for 10 min and centrifuged for another 5 min (6000 rpm, 4 °C). Each activity was performed with limited access to light. After centrifugation, the supernatant was filtered through syringe filters (0.22 µm PTFE). One cubic centimeter of the solution was added to one cubic centimeter of eluent and injected into the column (5 µL). Separation was performed using a WATERS Acquity UPLC HSS T3 chromatographic column (2.1 × 100 mm, 1.8 μm; Waters, Ireland). The flow of the mobile phase, which was Milli-Q water with 0.1% formic acid, was 0.25 cm^3^/min. The column thermostat was set at 25 °C, and the sample temperature was 4 °C. The spectrum was analyzed at a wavelength of 245 nm. The L-ascorbic acid content was calculated based on a calibration curve prepared for the vitamin C analytical standard (VWR Chemicals BDH Prolabo, Leuven, Belgium) (0.005–0.100 mg/mL). Duplicate analyses were performed for each sample.

#### 2.3.8. Determination of Total Polyphenol Content

A spectroscopic method was used to determine the total polyphenol content using Folin–Ciocalteu reagent [18]. Ninety-six-well plastic plates were used for the study, on which 10 µL of ethanol extract and 10 µL of distilled water were placed. Next, 40 µL of five-fold diluted Folin–Ciocalteu reagent was added to the solution and mixed. After 3 min, 250 µL of saturated sodium carbonate was added and then mixed again. Incubation was carried out at room temperature for 60 min without exposure to light. Absorbance was measured at 750 nm using a plate reader (Multiskan Sky, Thermo Electron Co., St. Louis, MO, USA). The analysis was performed in duplicate. The blank was prepared similarly, with the extract replaced by the extraction reagent. Quantitative analysis was based on a calibration curve for chlorogenic acid. The assay was performed in triplicate.

#### 2.3.9. Antioxidant Activity

The determination of antioxidant activity was performed using a spectrophotometric method, which involves assessing the ability to reduce the cation radical 2,2-azinobis(3-ethylbenzothiazoline-6-sulfonate) (ABTS^•+^) and using the iron III reduction method [18]. The assay was performed in triplicate.

Preparation of the ABTS^•+^ reagent solution

First, 0.0384 g of the ABTS cation radical was weighed into a 10 cm^3^ volumetric flask using an analytical balance. Then, 0.0066 g of K_2_S_2_O_8_ was added, and the volume was topped up with water to 10 cm^3^. The prepared solution was mixed and placed in the refrigerator for 12 h.

Preparation of the ABTS^•+^ stock solution

First, 1 cm^3^ of the ABTS^•+^ stock solution was measured into a 100 cm^3^ beaker, topped up to the final volume with an 80% ethanol solution, and mixed thoroughly. The working solution was prepared immediately before analysis. The absorbance of the solution, prepared as described and measured at a wavelength of 734 nm, was between 0.680 and 0.720.

Determination of Antioxidant Capacity Against ABTS Radicals

Here, 96-well plastic plates were used to determine the antioxidant activity. The analyte solution was diluted 5 times. Then, 10 μL of the extract was placed on the plates, and 250 μL of the radical solution was added, mixed, and the absorbance was measured. The absorbance for ABTS was recorded after 6 min at a wavelength of 734 nm. The antioxidant capacity was determined using the decrease in absorbance of the radical solution in the presence of an antioxidant and expressed in mg of Trolox per 1 g of dry matter.

Determination of Antioxidant Capacity as the Reduction of Iron (III)

To determine the reduction power of iron (III) ions by the analyte, 25 μL of the extract, 50 μL of a 1% aqueous solution of potassium ferricyanide, and 75 μL of distilled water were pipetted into the well. All components were mixed and then placed in an incubator (INCU-Line ILS 10; VWR, Radnor, PA, USA) at a temperature of 50 °C. After 20 min, 50 μL of 10% trichloroacetic acid was added. Then, 100 μL of the reaction mixture was taken into an empty well, and 100 μL of distilled water and 20 μL of a 0.1% iron (III) chloride solution were added and mixed. After 10 min, the absorbance was measured at 700 nm against a blank sample. The value of the reduction power of iron (III) ions was expressed as mg of Trolox.

#### 2.3.10. Determination of Carotenoid Content

The spectrophotometric method was used to determine the content of carotenoids. The prepared samples were weighed on an analytical balance at 0.3–0.5 g (m_1_). Twenty milliliters of distilled water and 1 mL of Carrez I solution were added, and then mixed on a magnetic mixer. A similar operation was performed with the Carrez II solution. The prepared samples were left for 2 min; then, the solution was centrifuged at a central acceleration of 2000 g for 5 min. Next, the colorless solution above the precipitate was poured off. Twenty milliliters of acetone was added to the sediment, and the closed tube was stirred on a magnetic stirrer for about 3 min. The solution was then centrifuged at a central acceleration of 2000 g for 5 min. The yellow acetone solution from the precipitate was decanted into a separatory funnel. Twenty milliliters of petroleum ether were added using a cylinder. The mixture was shaken thoroughly, and the phases were allowed to separate. The acetone phase was subsequently discarded. Twenty milliliters of acetone was poured into a centrifuge tube and mixed on a magnetic stirrer. The solution was then centrifuged at a central acceleration of 2000 g for 5 min. The solution from the precipitate was carefully transferred to a separatory funnel. Three milliliters of distilled water was added to the solution, gently shaken, and allowed to separate the phases. The water–acetone phase was discarded. Approximately 1.5 g of anhydrous sodium sulfate was weighed into dry centrifuge tubes. The ether solution was added from the separator, poured into the centrifuge tube, mixed with a magnetic stirrer, and centrifuged at 2000 g for 5 min. In a 50 mL volumetric flask, the solution from the precipitate was carefully poured into the flask with the previous extract. Then, the volumetric flask was filled to the mark with the petroleum ether solution and then mixed thoroughly. The wavelength of the UV-VIS spectrophotometer was set to 450 nm, and the instrument was zeroed against petroleum ether. The absorbance of the solution with carotenoids was measured in a 1 cm thick cuvette. The carotenoid content was calculated as β-carotene using the following formula:

ρ(C_40_H_56_) = A_450_ ⸱ 4.00 ⸱ (m_2_/m_1_), (5)

where ρ(C_40_H_56_)—total carotenoid content in mg/kg; A_450_—absorbance of the petroleum ether extract; 4.00—average conversion factor determined based on the ring test, considering the average absorption coefficient of β-carotene in petroleum ether and the dilutions made during the analysis; m_1_—sample mass in g; and m_2_—mass of ether extract, considering the density of the solvent.

#### 2.3.11. Microstructure

A Phenom XL scanning electron microscope (Thermo Fisher Scientific, Waltham, MA, USA) was used at a voltage of 10 kV and a vacuum of 60 Pa. Samples measuring 10 × 10 mm were cut from dried oranges and attached to a metal plate using carbon disks. The samples prepared in this way were covered with gold Cressington 108 auto (Cressington Scientific Instruments UK, Watford, UK) and placed in a vacuum chamber. Microscopic images of orange cross-sections were taken after 3 h of osmotic dehydration for convection-dried samples at 60× magnification.

#### 2.3.12. Organoleptic Assessment

The organoleptic assessment was performed for the samples in snack form. The samples were subjected to osmotic dehydration in different solutions and then dried using the convective method at 50 °C for 24 h. The dried orange slices were subjected to organoleptic evaluation by a group of 18 people. The respondents evaluated the following features: color, hardness, crispness, smell characteristic and foreign smells, taste characteristic and foreign taste, sourness, sweetness, general palatability and general quality. A 5-point scale was used, with the possibility of using an evaluation that was accurate to one decimal place, where 1 was the lowest possible score and 5 the highest.

#### 2.3.13. Statistical Analysis

The obtained data were statistically processed by one-way ANOVA using the Tukey test with the Statistica 13.3 program (StatSoft Polska Sp. z o.o., Cracow, Poland). All analyses were performed in triplicate, at least. Principal Component Analysis (PCA) and Pearson’s correlation analysis were conducted to evaluate the relationships between the selected variables. The PCA analysis considered the following properties after 3 h of osmotic dehydration in various osmotic agents: dry matter content (d.m.), water activity (aw), total soluble solids (°Brix), color (delta E), maximum force (Fmax), work, total sugar content, vitamin C content, polyphenol content (TPC), total carotenoid content (TCC), antioxidant activities (ABTS and Fe(III)).

## 3. Results and Discussion

### 3.1. The Effect of Unconventional Solutions on the Kinetics of the Osmotic Dehydration Process of Orange Slices

#### 3.1.1. The Effect of Unconventional Solutions on Weight Loss, Water Loss and Solid Gain During the Osmotic Dehydration Process of Orange Slices

**Figure 1:**
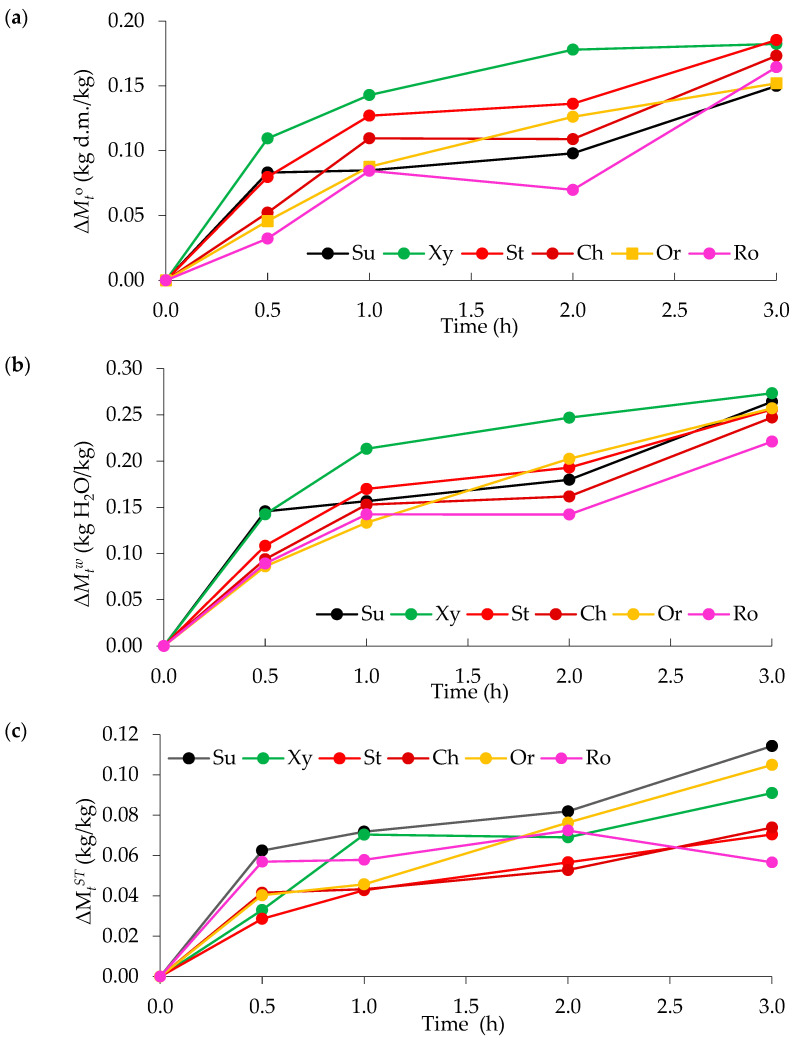
Osmotic dehydration kinetics: (**a**) Weight loss during 3 h of the osmotic dehydration process of oranges in different solutions; (**b**) Water loss during 3 h of the osmotic dehydration process of orange slices in different solutions; (**c**) Solid gain during 3 h of the osmotic dehydration process of orange slices in different solutions; Solutions: solution of sucrose (Su), xylitol (Xy), concentrates of cherry (Ch), strawberries (St), oranges (Or), and rosehip juice (Ro).

This work examined the weight loss caused by the dehydration time and the type of osmotic solution used, with values ranging from 0.03 to 0.19 kg d.m./kg (Figure 1a). The greatest weight loss was recorded in the sample dehydrated in a strawberry concentrate solution (0.19 kg d.m./kg) for 3 h. The sample dehydrated in a xylitol solution (0.18 kg d.m./kg) after 3 h had the same value after dehydration for 2 h, showing saturation with the osmotic solution. Samples dehydrated after 3 h had the highest values (0.15–0.19 kg d.m./kg), with no statistically significant differences between them (Table S1). The lowest value was observed in the sample dehydrated in the rosehip solution after 0.5 h (0.03 kg d.m./kg). After 0.5 h of dehydration, only the sample dehydrated in xylitol solution had a greater weight loss than the sample dehydrated in traditional sucrose solution (0.11 kg d.m./kg). After an hour of dehydration, the sample in the rosehip solution (0.08 kg d.m./kg) had the same mass loss as the sample in the sucrose solution; the remaining samples had a higher mass loss. After two hours, the slices dehydrated in rosehip solution (0.07 kg d.m./kg) had a lower parameter value than those dehydrated in sucrose (0.10 kg d.m./kg). The remaining samples had higher values. After dehydration for 3 h, the slices dehydrated in the orange concentrate solution had the same value (0.15 kg d.m./kg) as the patch dehydrated in sucrose (0.15 kg d.m./kg), while the remaining samples showed a higher weight loss. In almost every case, it can be seen that the weight loss increased with an increasing osmotic dehydration time. A different trend can be observed for the sample dehydrated in the rosehip solution, because the value after 2 h (0.07 kg d.m./kg) was lower than that after 1 h (0.08 kg d.m./kg). It can be concluded that the most intense mass loss occurred in the initial phases of dewatering, as it was observed that the differences in mass loss values became smaller as the process continued. Sebii et al. [27] noted that the reduction in the weight of the dehydrated pomegranate seeds varied remarkably during the 2 h of the osmotic dehydration in sucrose (from 6.25 to 19.35%), bitter orange juice (from 36.29 to 43.34%), apple juice (from 36.42 to 38.92%), and grape juice (from 43.67 to 50.73%) solutions. It was also shown that the sample in the sucrose solution had a smaller mass loss compared to the samples. Kowalska et al. [26] observed a 28–59% weight loss when dehydrating strawberries in fruit juices. The research results showed that the longer the dewatering time, the greater the mass loss. It was also noticed, similar to this study, that the greatest mass loss occurred in the initial phases of deodorization.

The type of osmotic solution and dehydration time influenced the water loss in oranges. The values ranged from 0.09 to 0.27 kg H_2_O/kg (Figure 1b). The greatest water loss occurred in orange slices dehydrated in xylitol solution for 3 h (0.27 kg H_2_O/kg), which was greater than the sample dehydrated in sucrose solution. It is worth noting that during the 3 h, the samples dehydrated in all solutions except rosehip did not differ statistically from the samples dehydrated in a traditional sucrose solution (Table S2). The samples in the rosehip, cherry, and orange solutions had the smallest water loss among all combinations within 0.5 h (0.09 kg H_2_O/kg). After 2 h of dehydration, the largest water loss was observed for the sample dehydrated in xylitol solution (0.25 kg H_2_O/kg), and the smallest was observed for that in the rosehip solution (0.14 kg H_2_O/kg). The samples in all solutions, except rosehip, had greater water loss compared to the traditional sucrose solution, but the difference between samples in the sucrose and strawberry solutions was not statistically significant. After an hour of dehydration, the largest water loss was observed in the sample dehydrated in the strawberry solution (0.17 kg H_2_O/kg), and the smallest was observed in the orange solution (0.13 kg H_2_O/kg). The orange samples in the rosehip, sucrose, and cherry solutions had statistically insignificant differences in water loss (Table S2) because they were in one homogeneous group, while the samples in the strawberry and xylitol solutions had higher water loss compared to the sample dehydrated in the sucrose solution. After 0.5 h of dehydration, the sample dehydrated in a solution of sucrose and xylitol had the highest water loss. In all cases, an increase in water loss with the duration of the process is noticeable, with the most intense water loss occurring in the initial phases of dewatering. In other studies, where strawberries were dehydrated in solutions of various fruit juices, the weight loss ranged from 3.9 to 7.9 g H_2_O/g. The strawberry samples dehydrated in solutions of chokeberry, strawberry, and cherry concentrate had a greater weight loss than those dehydrated in traditional sucrose solutions [26].

When analyzing the solid gain of orange samples during osmotic dehydration in various osmotic solutions, it was observed that the type of osmotic solution and the dehydration time impacted the obtained values (Figure 1c). The weight gain ranged from 0.11 kg/kg for the sample dehydrated in a sucrose solution for 3 h to 0.03 kg/kg for the sample dehydrated in a strawberry concentrate solution for 0.5 h. The highest weight gain was observed in the first phase of dehydration at 0.5 h; as the dehydration process continued, the differences were small. In the case of the dehydrated patch in rosehip and cherry solutions, statistical differences were visible only after 3 h of dehydration. The orange slice dehydrated for 3 h in an orange concentrate solution also had a high weight gain of 0.11 kg/kg, but this was statistically significantly lower than the sample dehydrated in a traditional sucrose solution (0.11 kg/kg) (Table S3). A similar trend was observed for the same samples during dewatering for 2 h. After an hour of dehydration, the sample in xylitol solution had the same weight gain as the sample dehydrated in traditional sucrose (0.07 kg/kg). After 0.5 h of dehydration, the samples dehydrated in the sucrose solution and rosehip concentrate solution had the highest mass increase. In the study conducted by Kowalska et al. [26], small differences in the weight gain values were observed for dehydrated strawberries in fruit juices. The values ranged from approximately 0.3 to 1 kg/kg. The highest rate of solid mass increase occurred at the initial stage of the process, regardless of the type of hypertonic solution.

#### 3.1.2. The Effect of Unconventional Solutions on the Effectiveness of the Osmotic Dehydration Process of Orange Slices

The effectiveness of the osmotic dehydration process of oranges in various solutions depended on the dehydration time and the type of osmotic solution used (Table 2). These values ranged from 1.56 kg/kg for an orange dehydrated in a rosehip solution for 0.5 h to 4.33 kg/kg for an orange dehydrated in a xylitol solution also for 0.5 h. Dehydration in the xylitol solution was most intense in the initial phases of dehydration. Another high value was recorded for the sample of orange dehydrated in a rosehip solution (3.96 kg/kg) and the orange dehydrated in a strawberry concentrate solution for 1 h. Osmotic dehydration in the xylitol and strawberry solutions was more effective than in the sucrose solution at all dehydration times. In the case of the sample dehydrated in xylitol solution, the most effective dehydration times were 0.5 h (4.33 kg/kg), 2 h (3.57 kg/kg), 1 h (3.03 kg/kg), and 3 h (3.02 kg/kg), with no statistical differences observed between 1 and 3 h. The sample’s dewatering efficiency in the strawberry solution increased with time. For the sample dehydrated in the cherry solution, the highest efficiency was at 1 h (3.52 kg/kg), followed by 3 h (3.46 kg/kg), 2 h (3.13 kg/kg), and 0.5 h (2.27 kg/kg). The differences between each time were not statistically significant. For the sample in the orange solution, the highest efficiency was achieved with dehydration for 1 hour (2.92 kg/kg), followed by 2 h (2.66 kg/kg), 3 h (2.45 kg/kg), and 0.5 h (2.15 kg/kg). No statistically significant differences were recorded for these samples either. Orange dehydration in rosehip solution achieved the highest efficiency in the last dehydration stage of 3 h (3.96 kg/kg), followed by 1 hour (2.47 kg/kg). There were no statistical differences between dehydration times of 2 h (1.97 kg/kg) and 0.5 h (1.56 kg/kg). In research by Kowalska et al. [26], the authors also observed the influence of time and the type of solution used on the efficiency of strawberry dehydration.

### 3.2. The Effect of Unconventional Solutions on the Dry Matter Content, Water Activity and Total Soluble Solids of the Osmotic Dehydration Process of Orange Slices

Based on the obtained tests, the influence of each solution and the osmotic dehydration method on water activity was found. The sample values ranged from 0.892 to 0.942 (Table 3). Fresh orange had the highest water activity (0.942) because it was not osmotically dehydrated. After analyzing the results, it was noticed that the orange dehydrated in a solution of strawberry concentrate (0.914) and rosehip (0.927) had the highest water activity among all dehydrated oranges. These differences were not statistically significant. The lowest water activity was found for oranges dehydrated in solutions of sucrose, xylitol (0.900), and concentrate from cherries (0.904) and oranges (0.903). In the graph, we can see the differences between the solutions used, but they were not statistically significant. It can be concluded that these osmotic solutions were as effective as the traditional sucrose solution. The available literature also confirms the effect of water activity when using osmotic dehydration and various solutions [28]. The authors used vacuum osmotic dehydration on tomatoes, resulting in a decrease in water activity. It was found that maltodextrin solutions with sodium chloride reduce this parameter more than maltodextrin solutions with traditional sucrose. Although the raw material and dewatering method were different, the water activity values were similar, ranging from 0.980 to 0.920. During pulsed osmotic dehydration in a vacuum of green figs, a reduction in water activity from 0.93 ± 0.02 to 0.91 ± 0.01 was observed by de Mello et al. [29]. In the research of Kowalska et al. [26], where strawberries were dehydrated in osmotic solutions obtained from fruit concentrates (sucrose, chokeberry, strawberry, cherry), the water activity was within the range of 0.95–0.99.

**Figure 2:**
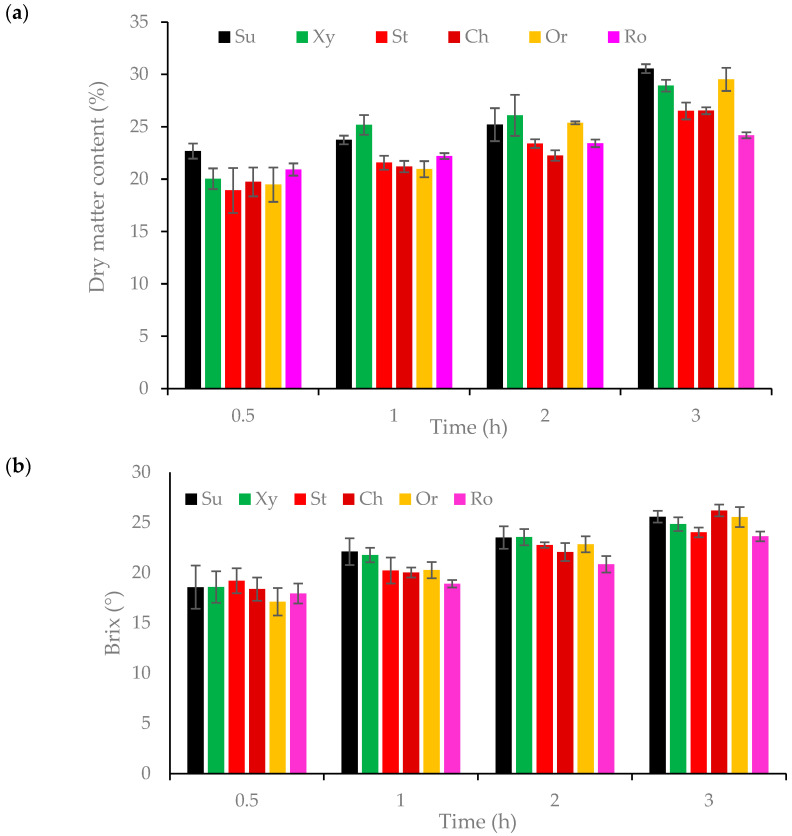
Properties of orange slices after a 3 h dehydration process: (**a**) Dry matter content of orange slices in different solutions; (**b**) Total soluble solids in orange slices in different solutions. Solutions: solution of sucrose (Su), xylitol (Xy), and concentrates of cherry (Ch), strawberries (St), oranges (Or), and rosehip juice (Ro).

The use of the osmotic dehydration process in various osmotic solutions and time influenced the dry matter content. The values ranged from 14.55% for a fresh sample to 30.56% for a sample dehydrated for 3 h in a sucrose solution (Figure 2a). An increasing trend in the values was observed, with the highest increases seen in samples after 3 h of dewatering. Analyzing the raw material after the dehydration process, the highest dry matter content was found in oranges dehydrated in sucrose solution (30.56%). The raw material dehydrated in the orange concentrate solution had the highest value, with a difference of only 1.03 g by mass. Another solution in which water was equally well removed from the raw material was xylitol, with a difference of 1.64 g compared to the sucrose solution. The differences between the orange concentrate solution and xylitol were not statistically significant, nor were the differences between the strawberry and cherry concentrate solutions (Table S4). Oranges dehydrated in a sour cherry concentrate solution had the lowest dry substance content (23.33%).

Additionally, it was observed that after 2 h of deodorization, the sample dehydrated in xylitol solution had the highest dry matter content (26.09%), followed by sucrose (25.20%). However, these differences were not statistically significant. The lowest dry substance content after 2 h was for samples dehydrated in cherry concentrate (22.25%). When analyzing the results obtained after osmotic dehydration for 1 hour, it can be seen that the highest dry substance content was in the sample dehydrated in the xylitol solution (25.18%), while the lowest was in the orange juice solution (20.95%). After osmotic dehydration for 30 min, the orange had the highest dry matter value in the sucrose solution (22.68%) and the lowest in the strawberry solution (18.92%). There were no statistically significant differences between the remaining solutions. Observing the obtained results, it can also be seen that all the samples after the dehydration process at all times had a higher dry substance content compared to the fresh orange slice. It can also be observed that the length of time affects the increase in the dry substance content of each osmotic solution used. In the available scientific studies in which strawberries were dehydrated in solutions of chokeberry, cherry, and strawberry concentrates, the dry matter content ranged from 20 to 38% [26].

The total soluble solids is the ratio of sugar content to water content, when sugar is dissolved in it. The use of the osmotic dehydration process with various osmotic solutions and times had an impact on the tested parameter. The values ranged from 10.17 to 26.2 °Brix (Figure 2). The highest °Brix value was observed for orange dehydrated for 3 h in a cherry solution (26.2 °Brix), followed by the sucrose solution (25.6 °Brix). The differences between them were not statistically significant (Table S5). The smallest one is in the solution obtained from strawberry concentrate (24.0 °Brix) and wild rose (23.6 °Brix). These differences were also not statistically significant. Analyzing the results obtained for the samples after dehydration for 2 h, the °Brix value is highest in the raw material dehydrated in a solution from cherry concentrate (22.1 °Brix), and lowest in wild rose (20.8 °Brix). Greater differences can be observed in the results after 2 and 3 h, and after 1 and 2 h of dehydration, compared to the differences between 0.5 and 1 h. Thus, it can be concluded that prolonged dehydration results in greater water removal and, consequently, a higher Brix value. The extract content after one hour of dehydration was highest for the sample in the sucrose solution (22.1 °Brix) and lowest in the rosehip solution (18.9 °Brix). Analyzing the obtained results, it is evident that osmotic dehydration after 30 min in each solution produced the same effect, as the differences between the °Brix values were not statistically significant. Looking at the prepared data, one can see a significant increase in this parameter with regard to the fresh raw material (10.17 °Brix). Additionally, the dewatering time affects the increase in the °Brix value for the raw material tested in each of the solutions. The research of Sebii et al. [27] focused on how the extract changes in solutions of sucrose, bitter orange juice, grapes, and apples before and after osmotic dehydration. A decrease in the value was shown, from 0.948 to 0.906, for bitter orange juice, confirming that sugar was transferred to the raw material and caused an increase in this parameter in the tested sample.

### 3.3. The Effect of Unconventional Solutions on the Color of the Osmotic Dehydration Process of Orange Slices

**Figure 3:**
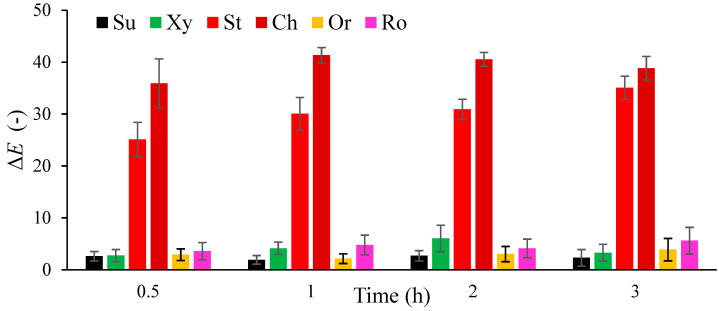
Total color difference (Δ*E*) of orange slices in a solution of sucrose (Su), xylitol (Xy), concentrates of cherry (Ch), strawberries (St), oranges (Or), and rosehip juice (Ro) during the 3 h the dehydration process.

The results for the total color differences, as the difference in the color of the raw material before and after osmotic dehydration in various solutions, is presented in Figure 3. The obtained results ranged from 3.0 for the fresh sample to 41.3 for the sample dehydrated for 2 h in the cherry solution. After processing the results, it was noticed that the raw material dehydrated in a solution of sucrose and orange concentrate did not differ from the fresh sample at all dehydration times. Orange slices dehydrated in xylitol and rosehip solutions also did not show significant differences. In the case of the sample in xylitol solution, a greater difference was noted after 2 h (6.0) and after 1 h (4.1) of dehydration than after 3 h (3.3). The greatest difference was observed for an orange slice dehydrated in a cherry concentrate solution (38.8), followed by a strawberry concentrate (35.1), for 3 h. In the case of the sample dehydrated in strawberry juice, the value of the total color difference increased proportionally with the dehydration time, from 25.1 to 35.1, but the values between 1 and 2 h of the process were not statistically significant (Table S6). In the research of Sebii et al. [27], there were higher values for the color parameters in dewatered raw material, which naturally differs in color from the osmotic solutions used in this process. In other scientific studies, the values of the absolute color difference were investigated for samples dehydrated in various osmotic solutions. In the case of strawberries, sucrose had the lowest values, followed by strawberry, cherry, and chokeberry juice [26].

### 3.4. The Effect of Unconventional Solutions on the Texture of the Osmotic Dehydration Process of Orange Slices

The texture of dehydrated oranges after 3 h of the process was tested to determine the force and work required to achieve 50% deformation of the osmotically dehydrated orange. Based on the results obtained, it is possible to determine the impact on the force value through the use of various solutions and the dehydration process. The obtained values ranged from 0.34 to 0.72 (Table 4). The tests showed that the highest force parameter to deform 50% of the raw material was obtained for oranges dehydrated in a strawberry concentrate solution (0.72 N), indicating that these samples were the hardest. It was also noted that the hardness of the sample dehydrated in rosehip solution (0.60 N) was closest to that of a fresh orange (0.65 N). It can be concluded that they were the same because the differences were not statistically significant (Table 4). All mentioned samples have a higher strength parameter than an orange osmotically dehydrated in a sucrose solution. The lowest force value needed to deform the orange by 50% was obtained for the raw material osmotically dehydrated in xylitol solutions (0.36 N) and cherry concentrates (0.34 N). The differences between them were not statistically significant (Table 4).

Based on the obtained research, it was noticed that the work necessary to achieve the 50% deformation of an osmotically dehydrated orange is similar to the force value, as work is the product of force and distance. It is also possible to observe the influence of the dehydration process and the type of solution on the work value. The results ranged from 0.25 to 0.6 mJ (Table 4). The highest value was observed for fresh orange (0.6 mJ), followed by wild rose (0.58 mJ) and strawberry (0.56 mJ). Oranges dehydrated in xylitol solutions (0.31 mJ) and orange concentrate (0.27 mJ) had a lower value than the sample dehydrated in a traditional sucrose solution (0.4 mJ). However, the lowest work value necessary to obtain 50% orange deformation was for the sample osmotically dehydrated in the cherry solution (0.25 mJ).

Barragán-Iglesias et al. [30] assessed the textures of papaya fruit dried by convection using a calcium hydroxide solution and osmotic dehydration. The texture profile analysis was carried out on fresh and convection-dried papaya at 70 °C without pre-treatment, as well as those pre-treated with calcium solutions and osmotic dehydration. The texture (force) for fresh raw materials was 8.64 ± 2.18 N, while with the osmotic application, it was 26.3 ± 5.50 N. The tests available in the literature have higher values, but they involve the complete deformation of another raw material that is subjected to two processes aimed at removing water. However, in studies involving the osmotic dehydration of pomegranate seeds in different osmotic solutions, more consistent results were observed. Their hardness before dewatering was 0.66 ± 0.09 N, but after dewatering, it decreased. In the bitter orange juice solution, it was 0.34 ± 0.04 N; in the apple juice solution, 0.55 ± 0.12 N; and in the grape juice solution, 0.53 ± 0.09 N [27].

### 3.5. The Effect of Unconventional Solutions on the Sugar Content of the Osmotic Dehydration Process of Orange Slices

The total sugar content is the sum of sucrose, glucose, and fructose, as determined in the research material. After analyzing the results, it was found that osmotic dehydration significantly increased the sugar content in the orange. This is due to the process’s mechanism, which aims to equalize the concentration gradient between the osmotic solution and the dehydrated raw material. The results ranged from 4.39 to 48.22 g/100 g (Table 5). The lowest sugar content was found in oranges dehydrated in xylitol solution (4.39 g/100 g), which is naturally characterized by a low sugar content and very low caloric value. Oranges dehydrated in orange concentrate solution had the highest sugar content (48.22 g/100 g). The raw material dehydrated in cherry (45.5 g/100 g) and strawberry (45.64 g/100 g) solutions had a higher content than that dehydrated in sucrose solution (43.92 g/100 g), but the differences were small. However, the dehydration of orange in the rosehip solution (43.71 g/100 g) behaved similarly to that in the sucrose solution (43.92 g/100 g d.m.), as the differences in the values were not statistically significant (Table 5).

The sucrose content in the samples ranged from 0.72 to 41.79 g/100 g d.m. (Table 5). The highest sucrose content was found in the orange slice osmotically dehydrated in sucrose solution (41.41 g/100 g d.m.) and also in the orange solution (41.79 g/100 g d.m.). The predominance of sucrose in the sucrose solution is evident on the graph, but it was not statistically significant. Next, the orange was dehydrated in rosehip solution (34.65 g/100 g d.m.). There were no significant statistical differences between orange slices dehydrated in a solution of strawberry concentrate (24.69 g/100 g d.m.) and cherry concentrate (24.56 g/100 g d.m.). An important finding is that the orange dehydrated in xylitol solution (0.72 g/100 g d.m.) had a lower sucrose content than the fresh slice (2.41 g/100 g d.m.).

The glucose content was also tested, and the results ranged from 0.77 to 9.22 g/100 g d.m. (Table 5). The highest value was found in the orange slice dehydrated in the strawberry concentrate solution (9.22 g/100 g d.m.), followed by the cherry concentrate solution (6.68 g/100 g d.m.), rosehip (3.59 g/100 g d.m.), and orange (2.27 g/100 g d.m.). The glucose content in fresh orange was 1.54 g/100 g d.m., while the patch dehydrated in xylitol solution had an even lower value (1.21 g/100 g d.m.) and the lowest value was found in the sucrose solution (0.77 g/100 g d.m.). Similarly, the fructose content was tested for all samples. The fructose content ranged from 1.75 to 13.86 g/100 g d.m. The highest value was found in an orange slice dehydrated in a solution of cherry concentrate (13.86 g/100 g d.m.) and strawberries (11.74 g/100 g d.m.). No statistical differences were noticed between them. Subsequently, these were samples dehydrated in rosehip solution (5.47 g/100 g d.m.). A slice of fresh orange had a glucose content of 2 g/100 g d.m.; a lower content was in the xylitol solution (2.47 g/100 g d.m.) and the lowest was in the sucrose solution (1.75 g/100 g d.m.). The available literature contains studies that confirm an increase in the total sugar content through osmotic dehydration in a sucrose solution. This was also observed in cherries, which, after dehydration, went from no sucrose to 2.5–19.6 g/100 g d.m., depending on the type of raw material. The glucose content showed both increasing and decreasing tendencies, while the fructose content increased [31]. Other studies have shown that the higher the sugar content in the osmotic solution, the higher the content in the final product. These studies were carried out on apples at various concentrations of sucrose [32].

### 3.6. The Effect of Unconventional Solutions on the Bioactive Compounds of the Osmotic Dehydration Process of Orange Slices

#### 3.6.1. Vitamin C Content

This study also determined the vitamin C content in oranges and observed that the type of osmotic solution used impacted its amount. The values ranged from 26.78 to 80.27 mg/100 g d.m. (Table 6). The highest vitamin C value was found in the sample dehydrated in a rosehip concentrate solution (80.27 mg/100 g d.m.), followed by a strawberry concentrate solution (62.32 mg/100 g d.m.). In both cases, the vitamin content was much higher than that of fresh orange (42.73 mg/100 g d.m.). In the slice dehydrated in the rosehip solution, the value was almost twice as high as that in the fresh raw material. The vitamin C content in an orange slice dehydrated in a solution from concentrate (47.67 mg/100 g d.m.) was higher compared to the fresh sample, but it was not statistically significant. The raw material dehydrated in each fruit concentrate solution had a higher vitamin C content than the orange dehydrated in a traditional sucrose solution. The lowest content of vitamin C was in orange slices dehydrated in sucrose solution (26.91 mg/100 g d.m.) and xylitol (26.91 mg/100 g dry matter), which is proven by the fact that it does not occur naturally in these solutions, unlike the others.

A decrease in vitamin C content was also observed during the osmotic dehydration of strawberries in polyol solutions. The process caused a significant decrease regardless of the type of polyols. After dehydration, the vitamin C content ranged from 22% to 43%. In fresh strawberries, the amount of vitamin C was 1.79 ± 0.04 mg/g d.m., while in the osmotically dehydrated material, this amount decreased to 0.39–0.77 mg/g d.m. [18]. In other studies, where strawberries were dehydrated in fruit juice solutions, the vitamin C content was also the highest in fresh fruit. Among the solutions used, the highest vitamin C value was found in fruits dehydrated in a strawberry juice concentrate solution (220.48 ± 3.62 mg/100 g d.m.), and the lowest (65.60 ± 3.03 mg/100 g d.m.) was found in dehydrated strawberries in sucrose solution [26].

#### 3.6.2. Total Polyphenols Content

The content of polyphenols, i.e., organic compounds with antioxidant properties, was examined. The type of dehydration solutions used had an impact on the content of the tested substance. The values ranged from 1297 to 2909 mg/100 g d.m. (Table 6). The highest content was recorded for raw material dehydrated in a strawberry concentrate solution (2909 mg/100 g d.m.). Compared to fresh orange (2527 mg/100 g d.m.), the content of compounds was also much higher. The remaining samples had a lower polyphenol content than the fresh raw material. It was also noticed that an orange slice dehydrated in a cherry concentrate solution (2366 mg/100 g d.m.) had a value quite similar to that of a fresh orange (2527 mg/100 g d.m.). The raw material dehydrated in fruit concentrate solutions had a higher polyphenol content than the sample dehydrated in a traditional sucrose solution (1518 mg/100 g d.m.). The sample dehydrated in the xylitol solution had the lowest content of the tested compound (1297 mg/100 g d.m.). This may be due to the nature of polyols, which naturally do not contain polyphenolic compounds. Scientific studies showed that after dehydration, there was a slight decrease in polyphenols from 384.22 meq GA/100 g d.m. before dewatering to 341.31, 376.49, 256.03, and 314.10 meq GA/100 g d.m. for sucrose solution and bitter orange, apple, and grape juices, respectively. The difference was mainly due to the initial composition of the juices and the changes that took place during the process. The raw material in these studies was pomegranate seeds [27].

#### 3.6.3. Antioxidant Activity

The next study aimed to determine the antioxidant potential against the ABTS cationic acid for oranges. As with previous determinations, it was observed that the selected osmotic solutions impacted the final parameter results. The values ranged from 2.7 to 11.0 mg/g d.m. (Table 6). A trend similar to that observed in the case of polyphenol and vitamin C content was observed; namely, there was a higher ABTS content in fruit concentrate solutions compared to traditional sucrose (2.7 mg/g d.m.). Here, even the raw material in the xylitol solution had higher values (2.8 mg/g d.m.), but this was not statistically significant. The highest value was observed in an orange slice dehydrated in a solution of strawberry concentrate (11.0 mg/g d.m.), followed by rosehip (8.8 mg/g d.m.) and cherry (8.5 mg/g d.m.). They had a higher ABTS content compared to fresh orange (7.7 mg/g d.m.); however, for the slices dehydrated in the rosehip and cherry solution, the differences were not statistically significant.

In the available literature, a quality assessment was conducted regarding the ability of samples to capture radical cations. ABTS showed that after the dehydration process in selected osmotic solutions, the ABTS values increased compared to the raw material. Relatively small changes were noted after the use of saline solution (an average increase of 47.2%) [33]. Kowalska et al. [26] observed that strawberries treated with sucrose solution and cherry juice concentrate exhibited 24.4–34.2% and 19.4–33.7% lower antioxidant activity values than fresh strawberries, respectively. In the case of strawberries dried in chokeberry and strawberry juice concentrates, the values of both indicators were higher by 1.3–5%. In the research of Wiktor et al. [18], it was found that the raw material dehydrated in polyol solutions had a lower ABTS content than the fresh sample. The antioxidant potential with iron (III) reduction, i.e., the reducing power that also determines the antioxidant properties, was examined. The use of different solutions impacted the tested parameter. The results ranged from 0.4 to 6.9 mg/g d.m. (Table 6). The highest value was observed in an orange slice osmotically dehydrated in a strawberry concentrate solution (6.9 mg/g d.m.). This value was higher compared to fresh raw material (4.1 mg/g d.m.). The remaining samples had lower values, but the sample dehydrated in sucrose solution (2.3 mg/g d.m.) and cherry (3.1 mg/g d.m) had the closest resemblance to fresh orange. The differences between them were not statistically significant. The lowest values were recorded for samples dehydrated in xylitol (1.1 mg/g d.m.), rosehip (1.0 mg/g d.m.), and orange (0.4 mg/g d.m.) solutions. There were no statistical differences between them either. The available literature also notes that raw materials dehydrated in polyol solutions have a lower reducing power value than fresh samples. Compared to traditional sucrose, the values were very similar, but the solutions used included mannitol and sorbitol in different concentrations [18].

#### 3.6.4. Carotenoid Content

Carotenoids are natural plant pigments and antioxidants that play an important role in human health [34]. The content of carotenoids in dehydrated orange slices ranged from 15.84 to 31.41 mg/kg d.m. (Table 6). Their highest content was found in fresh orange (31.41 mg/kg d.m.). For samples dehydrated in solution, the highest content was in orange dehydrated in a rosehip concentrate solution (24.25 mg/kg d.m.), followed by a traditional sucrose solution (20.75 mg/kg d.m.). No statistical differences were observed between oranges dehydrated in xylitol solutions (16.90 mg/kg d.m.), cherries (19.67 mg/kg d.m.), and oranges (17.21 mg/kg d.m.). The sample in the strawberry concentrate solution had the lowest content of the tested compound (15.84 mg/kg d.m.). In the study by Bialik et al. [35], where osmotic dehydration was used before the convective drying of kiwi fruit, the pre-treated raw material had a higher carotenoid content when dehydrated in a sucrose and mannitol solution.

### 3.7. The Effect of Unconventional Solutions on the Microstructure of the Osmotic Dehydration Process of Orange Slices

**Figure 4:**
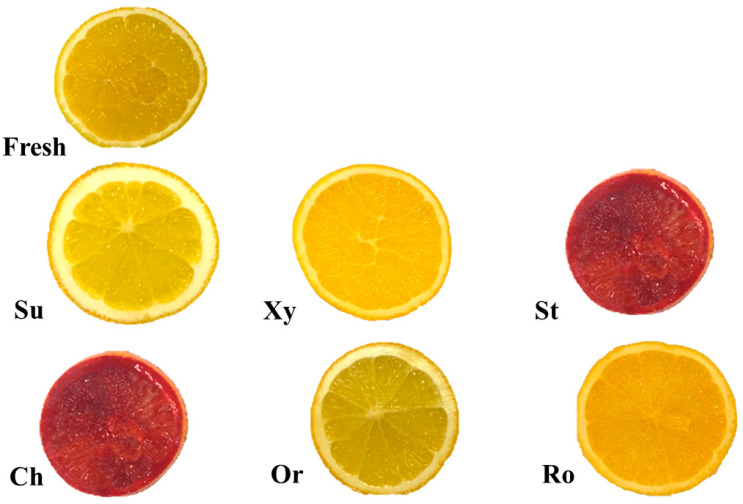
Digital pictures of osmotically dehydrated orange slices in a solution of sucrose (Su), xylitol (Xy), concentrates of cherry (Ch), strawberries (St), oranges (Or), and rosehip juice (Ro).

**Figure 5:**
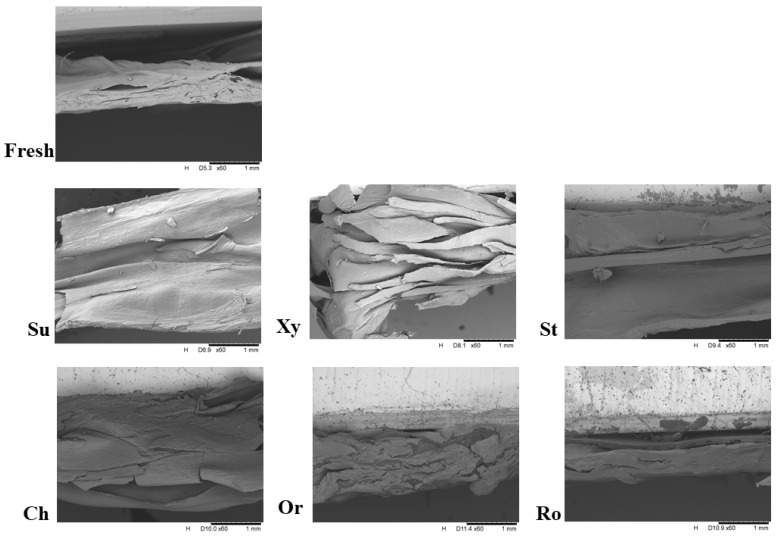
Scanning electron micrographs of osmotically dehydrated orange slices in a solution of sucrose (Su), xylitol (Xy), concentrates of cherry (Ch), strawberries (St), oranges (Or), and rosehip juice (Ro).

Figure 4 shows photographs of fresh orange slices and those after 3 h of osmotic dehydration in a solution of sucrose, xylitol, strawberry, cherry, orange, and rosehip juice concentrate. Significant color differences, namely a dark red shade, can be observed for samples dehydrated in the strawberry and cherry solution; this was related to the content of colored substances in these juices. Observations of the structure were also performed using a scanning electron microscope, and the photographs are shown in Figure 5. The microstructure of oranges that had previously been subjected to the convection drying process after 3 h of osmotic dehydration was examined. The effect of the solution used was visible in comparison to the non-dehydrated sample. It was noticed that the structure of the sample dehydrated in xylitol solution differed significantly from that of the fresh raw material. It was most disturbed, which may be due to the very low sugar content. This is probably due to the increased mass transfer area arising from the exclusion of intercellular air. In general, the microstructure of analyzed osmotically dehydrated orange slices can be described as featuring a rough surface with significantly more microscopic pores and cell ruptures. Similar observations were noted by Huang et al. [36].

The structure of the orange dehydrated in a rosehip and strawberry solution was most similar to the fresh sample. The raw material dehydrated in a traditional sucrose solution had a structure similar to the sample dehydrated in a cherry concentrate solution. Samples dehydrated osmotically through the process mechanism may have detached cell walls and a damaged structure due to cell shrinkage. Kowalska et al. [26] recorded similar observations, but in their study, the structural disturbance was much greater. This may be due to the raw material used, which was strawberries. Their thin walls could have contributed to the severe damage caused to the cell texture and intercellular space.

### 3.8. The Effect of Unconventional Solutions on the Organoleptic Assessment of the Osmotic Dehydration Process of Orange Slices

During the organoleptic evaluation, the following characteristics were assessed: color, hardness, crunchiness, the perceptibility of the characteristic smell and taste of orange, the perceptibility of a smell and taste foreign to orange, sourness, sweetness, general palatability, and overall quality assessment. The dried samples treated as snacks were submitted for sensory evaluation.

**Figure 6:**
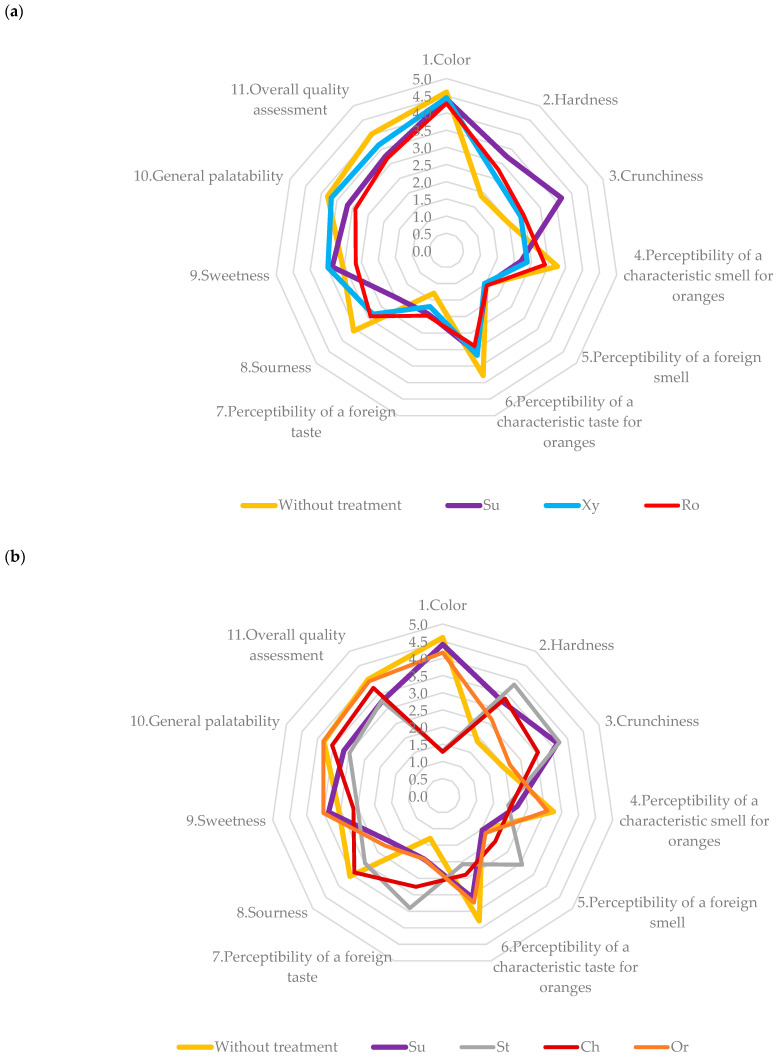
Organoleptic assessment of dehydrated orange slices in osmotic solutions (**a**) without treatment, sucrose (Su), xylitol (Xy), and rosehip juice (Ro); (**b**) concentrates of cherry (Ch), strawberries (St) and oranges (Or).

The obtained results are presented in Figure 6a (for the following solutions: sucrose, xylitol, and rosehip juice) and Figure 6b (for sucrose and concentrates solutions: strawberry, cherry, and orange), and the description of the results are presented according to this set of data. Also, all samples were compared to the untreated samples (without osmotic dehydration).

According to Figure 6a, it can be stated that in terms of color, the samples not subjected to the osmotic process and the oranges dehydrated in sucrose, xylitol, and rosehip juice solutions achieved similar results (4.5). In terms of hardness and crunchiness, the dried orange dehydrated in sucrose solution was the best, with 3.24 and 3.68 points, respectively. The orange without treatment was assessed as the worst, with 1.88 for hardness and 1.96 for crunchiness, respectively. The characteristic orange smell was most noticeable in the case of the non-dehydrated orange and the orange dehydrated in a rosehip juice solution, with 3.26 and 2.89 points, respectively. When using a solution of sucrose and xylitol during dehydration, these results were between 2.19 and 2.37 points. In the case of the samples treated with sucrose, xylitol, and rosehip juice, practically no foreign smell was detected (approx. 1.5 points). The sample with the most characteristic orange taste was the dried orange not subjected to osmotic dehydration (3.79 points). In this case, the perception of a foreign taste was rated the lowest (1.29 points). The least typical taste for oranges was characteristic of dried oranges dehydrated in rosehip juice solution (2.89). In terms of acidity, the highest assessment was given to the orange that was not dehydrated (3.57), then the orange dehydrated in rosehip juice solution (2.92), xylitol solution (2.83), and sucrose solution (2.03). In terms of sweetness, the highest scores were given to the orange dehydrated in xylitol solution (3.48), then in sucrose solution (3.36), then the orange not subjected to the osmotic process (3.05); the least sweet orange was dehydrated in rosehip juice solution (2.66). Both sucrose and xylitol are chemical compounds used in the food industry to sweeten food [18]. The use of their solutions during dehydration contributed to an increase in the sweetness level of the orange, despite the fact that these fruits themselves are characterized by a sweet and sour taste. Rosehip juice, due to its characteristic sour taste, should have increased the acidity of the orange; however, the addition of trehalose to the solution could have caused a decrease in its acidity and thus a lower assessment of this feature in relation to the non-dehydrated orange. However, this was still higher than the acidity assessment of the oranges dehydrated in sweeter solutions.

Osmotic dehydration in fruit concentrate solutions (Figure 6b) caused a change in color compared to undehydrated oranges. While it did not differ significantly for the orange concentrate solution, after dehydration in cherry and strawberry concentrate, the difference was significant. The value obtained for undehydrated slices was 4.61, while the values obtained for oranges dehydrated in strawberry and cherry juice concentrate were 1.31 and 1.28, respectively. The penetration of the colored osmotic solution into the orange tissue, as a result of which the samples darkened and acquired a red–burgundy color, was perceived negatively by the evaluators. Similar observations were also made by Samborska et al. [24]. According to the respondents, there was also an increase in hardness from 1.88 for the undehydrated sample to 3.85 for the sample dehydrated in strawberry juice concentrate. Also, the crunchiness ranged from 1.96 for an undehydrated sample to 3.74 for a strawberry solution. This was caused by the exchange of mass occurring during the osmosis process. Kowalska [37] proved that with an increase in the concentration of the osmotic solution, the penetration of the osmotic substance increases, which is caused by a decrease in water activity. As a result, there is an increase in mass loss, which makes the crunchiness and hardness more noticeable. Dusza and Hara [38] proved that in dried fruits previously subjected to osmotic dehydration, there is a decrease in mass loss, which makes the product harder than the product not subjected to dehydration. In addition, as the authors pointed out, osmotic dehydration accelerated convective drying, which resulted in a larger amount of water in the sample not subjected to the pre-treatment, which resulted in lower hardness. Osmotic dehydration caused a decrease in the perception of the taste and smell characteristic of oranges, and thus an increase in the perception of a foreign taste and smell, with the exception of samples dehydrated in orange juice concentrate, which, particularly in terms of smell, did not differ from slices not subjected to deodorization. The observed differences were caused by the addition of fruit concentrate solutions or sucrose, which caused a change in the taste and smell of orange slices. A small number of respondents stated that the orange dehydrated in the orange solution had a honey-like aftertaste. Others, however, believed that it was sweeter than the orange not subjected to dehydration. Respondents sensed a combination of the smell of dried orange with the predominant smell of the fruit additive. The greatest difference was observed in the case of the use of strawberry concentrate, which clearly gives an unfamiliar taste and smell of orange.

As a result of the penetration of sweet fruit juice concentrates, the perceived acidity decreased. The respondents noted that the orange dehydrated in a sucrose solution was the least acidic, while the undehydrated sample was the most acidic. During the osmosis process, sugar molecules penetrate the fruit and saturate it. As a result, the perception of acidity decreased (Figure 6b). An increase in the perception of sweetness was also observed in the case of the sucrose solution and the orange juice concentrate solution. Sucrose is a disaccharide consisting of fructose and glucose. Dehydration carried out in such a solution increases the content of fructose and glucose in the product [39], and consequently increases sweetness. However, it was different in the case of the osmotic solution, which was a cherry and strawberry juice concentrate. These fruits are quite sour, so dehydration in these concentrates limits the perceived sweetness. The lowest palatability and overall quality rating was characteristic of the dried orange dehydrated in a strawberry concentrate solution. The overall palatability was rated at 2.98, and the overall quality was rated at 3.28. This could be due to the largest difference in all of the tested characteristics, except for sourness. The strongly noticeable strawberry aroma and the remaining strawberry aftertaste reduced consumer interest in the product. The highest palatability and the highest overall quality rating were characteristic of oranges subjected to convective drying only and oranges dehydrated in an osmotic solution containing orange juice concentrate. The overall palatability value was at the level of 3.82, while the overall quality rating was 3.96. These were the oranges that were most similar to those expected by consumers. Samborska et al. [24] proved that mass transfer in the dehydrated material, which consists of a decrease in water content and the penetration of osmotic substances, affects color. This, in turn, together with other sensory characteristics, significantly affects the sensory assessment made by consumers.

### 3.9. The Principal Component Analysis

**Figure 7:**
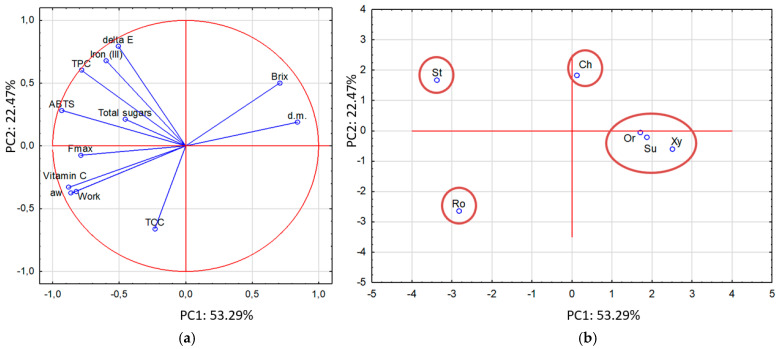
Principal Component Analysis (PCA): (**a**) PCA plot of two principal components, (**b**) score plot presenting analyzed samples dehydrated in different osmotic solutions (sucrose (Su), xylitol (Xy), concentrates of cherry (Ch), strawberries (St), oranges (Or) and rosehip juice (Ro)). in terms of PC1 vs. PC2. Properties: d.m.—dry matter content, aw—water activity, Brix—total soluble solids, delta E—color, Fmax—maximum force, work, total sugars, vitamin C, TPC—total polyphenols content, TCC—total carotenoid content, ABTS and Fe(III)—antioxidant activities.

Figure 7a illustrates the Principal Component Analysis (PCA) conducted on data obtained after a 3 h osmotic dehydration of oranges. The analysis aimed to identify differences and correlations among osmo-dehydrated oranges based on the type of osmotic solution used, which included sucrose (Su), xylitol (Xy), and concentrates of cherry (Ch), strawberry (St), orange (Or), and rosehip juice (Ro). The evaluation considered both physical properties (dry matter content, water activity, color, texture—maximum force and work) and chemical properties (total soluble solids, sugar content, vitamin C content, polyphenol content, carotenoid content, and antioxidant activity measured by ABTS and Fe(III)). The first principal component, PC1 (53.29%), explains the majority of the variability in the data, followed by PC2 (22.47%), together accounting for 75.76% of the variability in the properties of the dehydrated oranges.

The PCA results indicated that the antioxidant activities of osmo-dehydrated oranges (ABTS and Fe(III)) were positively correlated with the total polyphenol content (r^2^ = 0.905 and r^2^ = 0.833, respectively). This aligns with the well-established understanding that polyphenols significantly influence antioxidant activity [33,40]. Additionally, the total polyphenol content showed a positive correlation with the total color difference (r^2^ = 0.877). Osmotic solutions derived from fruit concentrates not only contain bioactive compounds but also affect the color of the osmotically dehydrated fruit, reflecting the source fruit’s characteristics. In our case, the strawberries and cherries concentrates gave the orange fruits a high total color change (see Figure 4), but also these solutions had a high amount of bioactive compounds.

What is more, the vitamin C content was negatively correlated with the dry matter content (r^2^ = −0.840), which means that a higher content of dry matter related to solid gain results in a lower content of vitamin C in the sample. According to the mechanism of the osmotic dehydration process, in two-directional mass transfer, water with solutes (e.g., vitamin C, which easily solutes in water) from the materials goes to osmotic solutions; meanwhile, the osmotic solutions penetrate the plant tissue, resulting in a solid gain [10]. This means that a higher solid gain and higher dry matter content result in a lower vitamin C content.

The solid gain also influences the textural properties of the osmodehydrated fruits. The total soluble solids (Brix) were negatively correlated with the texture parameter (work, r^2^ = −0.892), while the work necessary to compress the orange slice was positively correlated with the maximum force (Fmax, r^2^ = 0.937).

According to the score plot (Figure 7b), four distinct clusters can be identified. The first cluster includes three solutions: sucrose (Su), xylitol (Xy), and orange concentrate (Or); this indicates similar effects on the properties of dehydrated oranges. Cherry (Ch) and strawberry (St) concentrates, used as osmotic solutions, exhibit unique properties compared to the other osmotic agents, forming their own separate clusters. Additionally, rosehip juice (Ro) forms an independent cluster, highlighting its significant differences from the other samples. This analysis effectively highlights the differences in the effects of various osmotic solutions on the physical and chemical properties of dehydrated oranges.

## 4. Conclusions

The use of unconventional solutions, including xylitol, fruit juices from strawberry, orange and rosehip, as well as cherry concentrates, had a positive effect on the efficiency of the dehydration process in orange slices compared to the traditional sucrose solution. Oranges dehydrated in a solution of rosehip, strawberry, and orange juice were characterized by a higher vitamin C content than the fresh raw material. In the case of polyphenols and antioxidant potential, the highest values were observed for samples dehydrated in strawberry concentrate. However, most carotenoids were observed in samples dehydrated in the rosehip solution. Orange slices dehydrated in strawberry and cherry concentrate were dark red, while the remaining samples had a color similar to the raw material. A similar structure was observed in the control sample for oranges dehydrated in a solution of strawberries and wild rose. The use of unconventional solutions influenced the tested parameters of the physicochemical properties of oranges compared to the fresh sample and the sample dehydrated in a traditional sucrose solution.

Among the unconventional solutions used in the study, the xylitol solution was the best in terms of the organoleptic evaluation. The overall quality and palatability of the orange dehydrated in this solution were the highest compared to the other solutions. Moreover, the results obtained for this sample were the closest to the results of the orange not subjected to dehydration. The xylitol solution is therefore recommended for the osmotic dehydration of oranges due to its increased sweetness and lack of influence on the change in the taste and smell of oranges. The use of unconventional osmotic solutions offers great opportunities for the food industry in terms of creating new products. Their use, apart from being a sucrose substitute, enables the shaping of sensory and color characteristics. Nevertheless, this effect may vary due to the fruit’s structure and composition. More research is needed to verify the possibility of enhancing fresh products and their bioactive compounds with the use of unconventional osmotic solutions.

## Figures and Tables

**Table 1:** Characteristics of oranges before the osmotic dehydration process.

Measurement and Unit	Mean ± Standard Deviation
Dry matter (%)	14.55 ± 1.23
Water activity (-)	0.942 ± 0.007
Total soluble solids (°Brix)	10.17 ± 0.40
L*	49.59 ± 2.39
a*	1.67 ± 0.32
b*	22.78 ± 2.87
Texture–F_max_ (N)	0.65 ± 0.05
Texture–(work) surface area (mJ)	0.60 ± 0.12
Sugars content	
Sacharose (g/100 g d.m.)	2.41 ± 0.03
Glucose (g/100 g d.m.)	1.54 ± 0.02
Fructose (g/100 g d.m.)	4.22 ± 0.12
Vitamin C content (mg ascorbic acid/100 g d.m.)	42.73 ± 0.22
Total polyphenol content (mg chlorogenic/100 g d.m.)	2527 ± 63
Antioxidant activity against ABTS (mg TE/g d.m.)	7.7 ± 0.2
Reduction of iron (III) (mg TE/g d.m.)	4.1 ± 0.1
Carotenoid content (mg/kg d.m.)	31.41 ± 0.26

**Table 2:** The efficiency of the osmotic dehydration process in oranges in a solution of sucrose (Su), xylitol (Xy), concentrates of cherry (Ch), strawberries (St), oranges (Or), and rosehip juice (Ro) for 3 h.

Osmotic Solution	Time (h)			
	0.5	1	2	3
Su	2.32 ± 0.51 ^ab.A 1^	2.19 ± 0.25 ^a.A^	2.25 ± 0.45 ^ab.A^	2.33 ± 0.24 ^a.A^
Xy	4.33 ± 0.44 ^c.B^	3.03 ± 0.14 ^abc.A^	3.57 ± 0.21 ^c.AB^	3.02 ± 0.31 ^ab.A^
St	3.91 ± 1.16 ^bc.A^	3.96 ± 0.39 ^c.A^	3.41 ± 0.10 ^c.A^	3.64 ± 0.15 ^ab.A^
Ch	2.27 ± 0.27 ^ab.A^	3.52 ± 0.28 ^bc.A^	3.13 ± 0.61 ^bc.A^	3.46 ± 0.90 ^ab.A^
Or	2.15 ± 0.59 ^a.A^	2.92 ± 0.48 ^abc.A^	2.66 ± 0.14 ^abc.A^	2.45 ± 0.37 ^a.A^
Ro	1.56 ± 0.11 ^a.A^	2.47 ± 0.60 ^ab.A^	1.97 ± 0.17 ^a.A^	3.96 ± 0.53 ^b.B^

^1^ Mean values ± standard deviation. Values marked with the same letter symbols in columns (^a–c^) or rows (^A–B^) indicate no statistically significant differences (*p* < 0.05).

**Table 3:** Water activity of fresh and dehydrated oranges in a solution of sucrose (Su), xylitol (Xy), concentrates of cherry (Ch), strawberries (St), oranges (Or), and rosehip juice (Ro).

Osmotic Solution	Water Activity (-)
Fresh	0.942 ± 0.070 ^b 1^
Su	0.892 ± 0.013 ^a^
Xy	0.900 ± 0.015 ^a^
St	0.914 ± 0.010 ^ab^
Ch	0.904 ± 0.002 ^a^
Or	0.903 ± 0.001 ^a^
Ro	0.927 ± 0.005 ^ab^

^1^ Mean values ± standard deviation. Values marked with the same letter symbols in columns (^a–b^) indicate no statistically significant differences (*p* < 0.05).

**Table 4:** The texture of dehydrated oranges in a solution of sucrose (Su), xylitol (Xy), concentrates of cherry (Ch), strawberries (St), oranges (Or), and rosehip juice (Ro).

Osmotic Solution	F_max_ (N)	Work as Surface Area (mJ)
Fresh	0.65 ± 0.05 ^bc 1^	0.60 ± 0.12 ^cd^
Su	0.53 ± 0.16 ^b^	0.40 ± 0.13 ^bd^
Xy	0.36 ± 0.09 ^a^	0.31 ± 0.09 ^ab^
St	0.72 ± 0.17 ^c^	0.56 ± 0.15 ^c^
Ch	0.34 ± 0.05 ^a^	0.25 ± 0.04 ^a^
Or	0.36 ± 0.10 ^a^	0.27 ± 0.07 ^ab^
Ro	0.60 ± 0.17 ^bc^	0.58 ± 0.18 ^c^

^1^ Mean values ± standard deviation. Values marked with the same letter symbols in columns (^a–d^) indicate no statistically significant differences (*p* < 0.05).

**Table 5:** The sugars of dehydrated oranges in a solution of sucrose (Su), xylitol (Xy), concentrates of cherry (Ch), strawberries (St), oranges (Or), and rosehip juice (Ro).

Osmotic Solution	Total Sugars (g/100 g d.m.)	Sucrose (g/100 g d.m.)	Glucose (g/100 g d.m.)	Fructose (g/100 g d.m.)
Fresh	8.17 ± 0.17 ^b 1^	2.41 ± 0.03 ^a^	1.54 ± 0.02 ^b^	4.22 ± 0.12 ^bc^
Su	43.92 ± 0.23 ^c^	41.41 ± 0.06 ^d^	0.77 ± 0.04 ^a^	1.75 ± 0.25 ^a^
Xy	4.39 ± 0.38 ^a^	0.72 ± 0.04 ^a^	1.21 ± 0.04 ^ab^	2.47 ± 0.45 ^ab^
St	45.64 ± 0.03 ^cd^	24.69 ± 0.14 ^b^	9.22 ± 0.24 ^f^	11.74 ± 0.12 ^d^
Ch	45.10 ± 1.82 ^cd^	24.56 ± 0.44 ^b^	6.68 ± 0.15 ^e^	13.86 ± 1.53 ^d^
Or	48.22 ± 0.21 ^d^	41.79 ± 0.06 ^d^	2.27 ± 0.13 ^c^	4.16 ± 0.00 ^abc^
Ro	43.71 ± 1.63 ^c^	34.65 ± 1.63 ^c^	3.59 ± 0.00 ^d^	5.47 ± 0.00 ^c^

^1^ Mean values ± standard deviation. Values marked with the same letter symbols in columns (^a–f^) indicate no statistically significant differences (*p* < 0.05).

**Table 6:** The content of vitamin C, total polyphenols, carotenoids and antioxidant activity (with ABTS radical and Iron (III) reduction) in a solution of sucrose (Su), xylitol (Xy), concentrates of cherry (Ch), strawberries (St), oranges (Or), and rosehip juice (Ro).

Osmotic Solution	Vitamin C (g/100 g d.m)	Total Polyphenols (mg chlorogenic acid/100 g d.m.)	Carotenoids (mg/kg d.m.)	ABTS (mg TE/d.m.)	Iron (III) Reduction (mg TE/g d.m.)
Fresh	42.73 ± 0.22 ^b 1^	2527 ± 63 ^cd^	31.41 ± 0.26 ^d^	7.7 ± 0.2 ^b^	4.1 ± 0.1 ^b^
Su	26.91 ± 2.09 ^a^	1518 ± 110 ^ab^	20.75 ± 1.29 ^bc^	2.7 ± 0.1 ^a^	2.3 ± 0.2 ^ab^
Xy	26.78 ± 0.9 ^a^	1297 ± 116 ^a^	16.90 ± 0.83 ^ab^	2.8 ± 0.1 ^a^	1.1 ± 0.4 ^a^
St	62.32 ± 3.12 ^c^	2909 ± 137 ^d^	15.84 ± 0.80 ^a^	11.0 ± 0.6 ^c^	6.9 ± 1.0 ^c^
Ch	40.68 ± 2.58 ^b^	2366 ± 120 ^c^	19.67 ± 0.83 ^ab^	8.5 ± 0.6 ^b^	3.1 ± 1.5 ^ab^
Or	47.67 ± 2.48 ^b^	1777 ± 62 ^b^	17.21 ± 0.19 ^ab^	3.4 ± 0.3 ^a^	0.4 ± 0.2 ^a^
Ro	80.27 ± 1.18 ^d^	1929 ± 95 ^b^	24.25 ± 1.85 ^c^	8.8 ± 0.4 ^b^	1.0 ± 0.1 ^a^

^1^ Mean values ± standard deviation. Values marked with the same letter symbols in columns (^a–d^) indicate no statistically significant differences (*p* < 0.05).

## Data Availability

The original contributions presented in this study are included in the article/supplementary material. Further inquiries can be directed to the corresponding authors.

## Supplementary Materials

The following supporting information can be downloaded at: https://www.mdpi.com/article/10.3390/foods14030468/s1, Table S1: Weight loss during the osmotic dehydration process of oranges in a solution of sucrose (Su), xylitol (Xy), concentrates of cherry (Ch), strawberries (St), oranges (Or), and rosehip juice (Ro); Table S2: Water loss during the osmotic dehydration process of orange slices in a solution of sucrose (Su), xylitol (Xy), concentrates of cherry (Ch), strawberries (St), oranges (Or), and rosehip juice (Ro); Table S3: Solid gain during the osmotic dehydration process of oranges in a solution of sucrose (Su), xylitol (Xy), concentrates of cherry (Ch), strawberries (St), oranges (Or), and rosehip juice (Ro); Table S4: Dry matter content of orange slices in a solution of sucrose (Su), xylitol (Xy), concentrates of cherry (Ch), strawberries (St), oranges (Or), and rosehip juice (Ro); Table S5: Total soluble solids orange slices in a solution of sucrose (Su), xylitol (Xy), concentrates of cherry (Ch), strawberries (St), oranges (Or), and rosehip juice (Ro); Table S6: Total color difference (ΔE) of orange slices in a solution of sucrose (Su), xylitol (Xy), concentrates of cherry (Ch), strawberries (St), oranges (Or), and rosehip juice (Ro).
