# Supplementary material for: The Effect of the Use of Unconventional Solutions for Osmotic Dehydration on Selected Properties of Fresh-Cut Oranges
Source: Foods. 2025 Feb 1;14(3):468. doi: 10.3390/foods14030468 (PMC11816818; doi:10.3390/foods14030468)
Supplement: Supplementary file 1 [file foods-14-00468-s001.zip › foods-3431940-supplementary.pdf]

foods

MDPI

Supplementary Materials

Table S1. Weight loss during the osmotic dehydration process of oranges in a solution of sucrose (Su), xylitol (Xy), concentrates of cherry (Ch), strawberries (St), oranges (Or), and rosehip juice (Ro).

Osmotic solution	Time (h)			
	0.5	1	2	3
Su	$0.08 \pm 0.03^{bc.A}$	$0.08 \pm 0.01^{a.A}$	$0.10 \pm 0.03^{ab.AB}$	$0.15 \pm 0.02^{a.B}$
Xy	$0.11 \pm 0.01^{c.A}$	$0.14 \pm 0.01^{c.B}$	$0.18 \pm 0.01^{c.C}$	$0.18 \pm 0.02^{a.C}$
St	$0.08 \pm 0.02^{bc.A}$	$0.13 \pm 0.01^{bc.B}$	$0.14 \pm 0.00^{bc.B}$	$0.19 \pm 0.01^{a.C}$
Ch	$0.05 \pm 0.01^{ab.A}$	$0.11 \pm 0.01^{abc.A}$	$0.11 \pm 0.02^{ab.A}$	$0.17 \pm 0.04^{a.B}$
Or	$0.04 \pm 0.02^{ab.A}$	$0.09 \pm 0.02^{ab.AB}$	$0.13 \pm 0.01^{b.BC}$	$0.15 \pm 0.03^{a.C}$
Ro	$0.03 \pm 0.01^{a.A}$	$0.08 \pm 0.03^{a.AB}$	$0.07 \pm 0.01^{a.B}$	$0.17 \pm 0.02^{a.C}$

Mean values ± standard deviation. Values marked with the same letter symbols in columns ($^{a-c}$) or rows ($^{A-C}$) indicate no statistically significant differences ($p<0.05$).

Table S2. Water loss during the osmotic dehydration process of orange slices in a solution of sucrose (Su), xylitol (Xy), concentrates of cherry (Ch), strawberries (St), oranges (Or), and rosehip juice (Ro).

Osmotic solution	Time (h)			
	0.5	1	2	3
Su	$0.14 \pm 0.02^{c.A}$	$0.16 \pm 0.01^{ab.A}$	$0.18 \pm 0.02^{bc.A}$	$0.26 \pm 0.01^{ab.B}$
Xy	$0.14 \pm 0.01^{c.A}$	$0.21 \pm 0.00^{c.B}$	$0.25 \pm 0.01^{d.C}$	$0.27 \pm 0.01^{b.D}$
St	$0.11 \pm 0.02^{ab.A}$	$0.17 \pm 0.01^{b.B}$	$0.19 \pm 0.00^{bc.B}$	$0.26 \pm 0.00^{ab.C}$
Ch	$0.09 \pm 0.01^{a.A}$	$0.15 \pm 0.01^{ab.B}$	$0.16 \pm 0.02^{ab.B}$	$0.25 \pm 0.03^{ab.C}$
Or	$0.09 \pm 0.02^{a.A}$	$0.13 \pm 0.01^{a.B}$	$0.20 \pm 0.01^{c.C}$	$0.26 \pm 0.02^{ab.D}$
Ro	$0.09 \pm 0.00^{a.A}$	$0.14 \pm 0.02^{ab.B}$	$0.14 \pm 0.01^{a.B}$	$0.22 \pm 0.01^{a.C}$

Mean values ± standard deviation. Values marked with the same letter symbols in columns ($^{a-d}$) or rows ($^{A-D}$) indicate no statistically significant differences ($p<0.05$).

Table S3. Solid gain during the osmotic dehydration process of oranges in a solution of sucrose (Su), xylitol (Xy), concentrates of cherry (Ch), strawberries (St), oranges (Or), and rosehip juice (Ro).

Osmotic solution	Time (h)			
	0.5	1	2	3
Su	$0.06 \pm 0.01^{c.A}$	$0.07 \pm 0.00^{c.AB}$	$0.08 \pm 0.01^{c.B}$	$0.11 \pm 0.01^{d.C}$
Xy	$0.03\pm 0.00^{ab.A}$	$0.07 \pm 0.00^{c.B}$	$0.07 \pm 0.00^{b.B}$	$0.09 \pm 0.01^{bc.C}$
St	$0.03 \pm 0.00^{a.A}$	$0.04 \pm 0.00^{a.B}$	$0.06 \pm 0.00^{a.C}$	$0.07 \pm 0.00^{a.D}$
Ch	$0.04 \pm 0.00^{b.A}$	$0.04 \pm 0.00^{a.A}$	$0.05 \pm 0.00^{a.A}$	$0.07 \pm 0.01^{ab.B}$
Or	$0.04 \pm 0.00^{b.A}$	$0.05 \pm 0.00^{a.A}$	$0.08 \pm 0.00^{bc.B}$	$0.11 \pm 0.01^{cd.C}$
Ro	$0.06 \pm 0.00^{c.A}$	$0.06 \pm 0.01^{b.A}$	$0.07 \pm 0.00^{bc.A}$	$0.06 \pm 0.00^{a.B}$

Mean values ± standard deviation. Values marked with the same letter symbols in columns ($^{a-d}$) or rows ($^{A-D}$) indicate no statistically significant differences ($p<0.05$).

Table S4. Dry matter content of orange slices in a solution of sucrose (Su), xylitol (Xy), concentrates of cherry (Ch), strawberries (St), oranges (Or), and rosehip juice (Ro).

**Osmotic solution**	**Time (h)**				
	**0**	**0.5**	**1**	**2**	**3**
Su	14.55 ± 1.23^{A}	22.68 ± 0.72$^{b.B}$	23.74 ± 0.41$^{bc.BC}$	25.20 ± 1.57$^{b.C}$	30.56 ± 0.42$^{d.D}$
Xy	14.55 ± 1.23^{A}	20.04 ± 0.99$^{ab.B}$	25.18 ± 0.94$^{c.C}$	26.09 ±1.96$^{b.CD}$	28.92 ± 0.56$^{c.E}$
St	14.55 ± 1.23^{A}	18.92 ± 2.15$^{a.B}$	21.57 ± 0.67$^{a.BC}$	23.40 ±0.41$^{ab.CD}$	24.64 ± 1.78$^{b.D}$
Ch	14.55 ± 1.23^{A}	19.73 ± 1.37$^{ab.B}$	21.20 ± 0.54$^{a.BC}$	22.25 ± 0.50$^{a.C}$	23.33 ± 2.48$^{a.D}$
Or	14.55 ± 1.23^{A}	19.47 ± 1.64$^{ab.B}$	20.95 ± 0.77$^{a.B}$	25.38 ± 0.14$^{b.C}$	29.53 ± 1.10$^{c.D}$
Ro	14.55 ± 1.23^{A}	20.92 ± 0.58$^{ab.B}$	22.21 ± 0.28$^{ab.BC}$	22.99 ±0.65$^{ab.CD}$	24.19 ± 0.29$^{b.D}$

Mean values ± standard deviation. Values marked with the same letter symbols in columns ($^{a-c}$) or rows ($^{A-D}$) indicate no statistically significant differences ($p<0.05$).

Table S5. Total soluble solids orange slices in a solution of sucrose (Su), xylitol (Xy), concentrates of cherry (Ch), strawberries (St), oranges (Or), and rosehip juice (Ro).

**Osmotic solution**	**Time (h)**				
	**0**	**0.5**	**1**	**2**	**3**
Su	10.17 ± 0.40^{A}	18.6 ± 2.2$^{a.B}$	22.1 ± 1.3$^{c.C}$	23.5 ± 1.1$^{b.CD}$	25.6 ± 0.6$^{bc.D}$
Xy	10.17 ± 0.40^{A}	18.6 ± 1.6$^{a.B}$	21.8 ± 0.7$^{bc.C}$	23.5 ± 0.8$^{b.D}$	24.8 ± 0.7$^{ab.D}$
St	10.17 ± 0.40^{A}	19.2 ± 1.2$^{a.B}$	20.2 ± 1.3$^{ab.B}$	22.8 ± 0.3$^{b.C}$	24.0 ± 0.5$^{a.C}$
Ch	10.17 ± 0.40^{A}	18.4 ± 1.2$^{a.B}$	20.0 ± 0.5$^{ab.C}$	22.1 ± 0.9$^{ab.D}$	26.2 ± 0.6$^{c.E}$
Or	10.17 ± 0.40^{A}	17.1 ± 1.4$^{a.B}$	20.3 ± 0.8$^{ab.C}$	22.8 ± 0.8$^{b.D}$	25.5 ± 1.0$^{bc.E}$
Ro	10.17 ± 0.40^{A}	17.9 ± 1.0$^{a.B}$	18.9 ± 0.4$^{a.B}$	20.8 ± 0.8$^{a.C}$	23.6 ± 0.5^{a,D}

Mean values ± standard deviation. Values marked with the same letter symbols in columns ($^{a-c}$) or rows ($^{A-E}$) indicate no statistically significant differences ($p<0.05$).

Table S6. Total color difference (ΔE) of orange slices in a solution of sucrose (Su), xylitol (Xy), concentrates of cherry (Ch), strawberries (St), oranges (Or), and rosehip juice (Ro).

**Osmotic solution**	**Time (h)**				
	**0**	**0.5**	**1**	**2**	**3**
Su	3.0 ± 2.0^{A}	2.6 ± 0.9$^{a.A}$	1.9 ± 0.8$^{a.A}$	2.7 ± 1.0$^{a.A}$	2.3 ± 1.6$^{a.A}$
Xy	3.0 ± 2.0^{A}	2.7 ± 1.2$^{a.A}$	4.1 ± 1.2$^{ab.AB}$	6.0 ± 2.6$^{b.B}$	3.3 ± 1.6$^{ab.A}$
St	3.0 ± 2.0^{A}	25.1 ± 3.3$^{b.B}$	30.1 ± 3.1$^{c.C}$	30.9 ± 2.0$^{c.C}$	35.1 ± 2.2$^{c.D}$
Ch	3.0 ± 2.0^{A}	35.9 ± 4.7$^{c.B}$	41.3 ± 1.5$^{d.C}$	40.6 ± 1.3$^{d.C}$	38.8 ± 2.3$^{d.BC}$
Or	3.0 ± 2.0^{A}	2.9 ± 1.1$^{a.A}$	2.1 ± 0.9$^{a.A}$	3.0 ± 1.5$^{a.A}$	3.9 ± 2.2$^{ab.A}$
Ro	3.0 ± 2.0^{A}	3.6 ± 1.7$^{a.A}$	4.8 ± 1.9$^{b.A}$	4.1 ± 1.8$^{ab.A}$	5.6 ± 2.6$^{b.A}$

Mean values ± standard deviation. Values marked with the same letter symbols in columns ($^{a-d}$) or rows ($^{A-D}$) indicate no statistically significant differences ($p<0.05$).
